# Targeted mutagenesis using the *Agrobacterium tumefaciens*-mediated CRISPR-Cas9 system in common wheat

**DOI:** 10.1186/s12870-018-1496-x

**Published:** 2018-11-26

**Authors:** Shujuan Zhang, Rongzhi Zhang, Guoqi Song, Jie Gao, Wei Li, Xiaodong Han, Mingli Chen, Yulian Li, Genying Li

**Affiliations:** 1 Crop Research Institute, Shandong Academy of Agricultural Sciences, 202 Gongyebei Road, Jinan, 250100 Shandong People’s Republic of China; 2 Key Laboratory of Wheat Biology and Genetic Improvement on North Yellow and Huai River Valley, Ministry of Agriculture, Jinan, 250100 Shandong People’s Republic of China; 3 National Engineering Laboratory for Wheat and Maize, Jinan, 250100 Shandong People’s Republic of China

**Keywords:** *Agrobacterium tumefaciens* transformation, CRISPR/Cas9, Gene editing, Protoplast, Wheat

## Abstract

**Background:**

Recently, the CRISPR/Cas9 system has been widely used to precisely edit plant genomes. Due to the difficulty in *Agrobacterium*-mediated genetic transformation of wheat, the reported applications in CRISPR/Cas9 system were all based on the biolistic transformation.

**Results:**

In the present study, we efficiently applied targeted mutagenesis in common wheat (*Triticum aestivum* L.) protoplasts and transgenic T0 plants using the CRISPR/Cas9 system delivered via *Agrobacterium tumefaciens*. Seven target sites in three genes (*Pinb, waxy* and *DA1*) were selected to construct individual expression vectors. The activities of the sgRNAs were evaluated by transforming the constructed vectors into wheat protoplasts. Mutations in the targets were detected by Illumina sequencing. Genome editing, including insertions or deletions at the target sites, was found in the wheat protoplast cells. The highest mutation efficiency was 6.8% in the *DA1* gene. The CRISPR/Cas9 binary vector targeting the *DA1* gene was then transformed into common wheat plants by *Agrobacterium tumefaciens*-mediated transformation, resulting in efficient target gene editing in the T0 generation. Thirteen mutant lines were generated, and the mutation efficiency was 54.17%. Mutations were found in the A and B genomes of the transgenic plants but not in the D genome. In addition, off-target mutations were not detected in regions that were highly homologous to the sgRNA sequences.

**Conclusions:**

Our results showed that our *Agrobacterium*-mediated CRISPR/Cas9 system can be used for targeted mutations and facilitated wheat genetic improvement.

**Electronic supplementary material:**

The online version of this article (10.1186/s12870-018-1496-x) contains supplementary material, which is available to authorized users.

## Background

Targeted mutagenesis plays an important role in functional genomics and crop improvement. The CRISPR/Cas9 system has been widely used to precisely edit plant genomes recently. In plants, the first report about the CRISPR/Cas9 system involved *Arabidopsis thaliana* [1, 2], followed by reports of its use in tobacco [1, 3], rice [4–8], soybean [9–11], sorghum [1], maize [12–15], tomato [16], barley [17], orange [18], watermelon [19], grape [20] and wheat [21–24]. The CRISPR/Cas9 system has developed rapidly and is regarded as a promising method for crop improvement. Transgenic plants with high mutation frequency in the targeted genes can be obtained by the CRISPR/Cas9 system, and the mutations can be inherited in the next generation stably.

Common wheat (*Triticum aestivum* L.) is one of the most widely grown crops in the world and a major component of the human diet. It has a large (17 Gb), complex polyploid genome with a high proportion of repetitive sequences (> 80%). These genomic traits create challenges for genetic and functional analyses in wheat. There are only a few reports of using CRISPR/Cas9-mediated targeted mutagenesis in common wheat. For example, the *TaMLO* mutants obtained by the CRISPR/Cas9 system show broad-spectrum resistance to powdery mildew. Transgenic wheat plants with mutations in the *TaMLO-A1* gene were generated by CRISPR/Cas9 technology [22]. Genes were edited in hexaploid bread wheat and tetraploid durum wheat, and mutants were generated with no detectable transgenes [25]. Zhang et al. [26] used CRISPR/Cas9 technology to generate *Taedr1* wheat plants by simultaneous modification of the three homoeologs of wheat *EDR1*, and the *Taedr1* plants were resistant to powdery mildew and did not show mildew-induced cell death. The amount of α-gliadins in the seed kernel can be efficiently reduced by CRISPR/Cas9 technology, providing bread and durum wheat lines with reduced immunoreactivity for gluten-intolerant consumers [23].

Because the genetic transformation of wheat is difficult, the above studies of the CRISPR/Cas9 system in wheat all used the biolistic transformation method. However, multiple-copy insertions can be produced by using the biolistic particle delivery method. It could also cause higher expression levels of the sgRNA and Cas9 protein. However, the *A. tumefaciens*-mediated method usually shows a higher single-site insertion frequency [27]. Such transgene insertions should be easier to locate in the plant genome [28]. Therefore, genetic analysis would be easier to carry out in the progeny of *A. tumefaciens*-mediated transgenic plants.

In this study, we report gene targeting of the CRISPR-Cas9 system in common wheat. Three target genes, the *Pinb* gene, granule-bound starch synthase gene (GBSS or waxy) and *DA1* gene, were selected. In common wheat, puroindoline a (*Pina*) and puroindoline b (*Pinb)* were the two major genes controlling grain hardness. It was known that a lack of *Pina* or a mutation in *Pinb* would cause wheat endosperm to be hard texture. In common wheat, the *Pinb* gene is located on chromosome 5DS. The starch composition of the grain has an important influence on wheat flour quality. Amylose is synthesized by granule-bound starch synthase (GBSS or waxy). In common wheat, *waxy* for each genome is encoded by *Wx-A1, Wx-B1* and *Wx-D1* located on chromosomes 7AS, 4AL (translocated from the original locus on 7BS) and 7DS, respectively [29]. The variants of wheat waxy proteins (Wx-A1, Wx-B1 and Wx-D1) have been used to determine the effect of waxy protein deficiencies on amylase content and starch pasting properties [30]. However, the frequencies of variation at the *waxy* locus are relatively low in modern wheat cultivars. DA1 is a ubiquitin receptor that functions as a negative regulator in seed and organ size control by restricting the period of cell proliferation [31]. The genome-edited wheat lines with *DA1* loss of function could be used to increase wheat yield and thousand-kernel weight. Thus, the *Pinb*, *waxy* and *DA1* genes offer potential for manipulating grain quality and size-related traits.

In this paper, we reported that the CRISPR/Cas9 system could efficiently conduct gene targeting in common wheat by *Agrobacterium tumefaciens*-mediated stable transformation. The gene targeting application of the *Agrobacterium*-mediated CRISPR/Cas9 system could become a promising biotechnology strategy for functional gene studies and facilitate the breeding efficiency of common wheat and other major crops.

## Methods

### Plasmid construction

The TaU6 (GenBank accession number: X63066.1) and TaU3 (GenBank accession number: X63065.1) promoters were synthesized and cloned into the pYPQ131D and pYPQ132C vectors to generate the pYPQ131D-TaU6 and pYPQ132C-TaU3 plasmids, respectively. The target gene oligonucleotides were annealed to form sgRNA duplexes and inserted into the pYPQ131D and pYPQ132C plasmids using *Bgl*II + *Bsm*BI, respectively. The sgRNAs of the *Pinb* gene were driven by the TaU6 promoter, and the sgRNAs of *waxy* and *DA1* were driven by the TaU3 promoter (Additional file 1: Table S1). PCR was performed to amplify the expression cassettes 131D-TaU6-sgRNAsc and 132-TaU3-sgRNAsc and then to produce the CRISPR/Cas9 construct with the binary vector pYLCRISPR/Cas9Pubi-B (GenBank accession number: KR029110.1) [6] using *Bsa*I digestion and ligated using T4 DNA ligase. The primers used in this experiment are listed in Additional file 1: Table S2. All constructed vectors were verified by sequencing.

### Protoplast isolation and transformation

We used the common wheat cultivar Fielder for protoplast isolation. The protoplasts were prepared from the fresh leaves of wheat seedlings as described by Shan et al. [32] with some modifications. The seeds were grown at 25 °C with a photoperiod of 16 h light and 8 h dark for 7–10 d. Fifteen to twenty fresh leaves were cut into small strips and incubated with 10 ml digestion solution (1.5% cellulose R10, 0.75% macerozyme, 0.6 M mannitol, 20 mM MES, 10 mM KCl, 10 mM CaCl_2_ and 0.1% BSA). The leaf strips were then incubated and digested at 28 °C for 4 h with gentle shaking (50 rpm) in the dark. After enzymatic digestion, W5 solution (2 mM MES, 154 mM NaCl, 125 mM CaCl_2,_ 5 mM KCl) was added to release the wheat protoplasts, and the strips were washed 3 times. The protoplasts were resuspended in MMG solution (0.4 M mannitol, 15 mM MgCl_2,_ 4 mM MES). The constructed plasmids were then transformed into wheat protoplasts through the PEG-mediated method. Finally, the wheat protoplasts were resuspended in 1 ml of W5 solution and incubated at 25 °C in the dark for 48–72 h.

### *Agrobacterium tumefaciens*-mediated common wheat transformation

After sequencing of the target sites, the binary vector was transformed into the wheat cultivar Fielder by *Agrobacterium tumefaciens*-mediated transformation [20, 33]. Briefly, wheat spikes were collected at anthesis, harvested 14–16 days post-anthesis (DPA) and sterilized with 75% ethanol for 30 s, then with 1% sodium hypochlorite (NaClO) for 15 min, and finally rinsed 5 times with sterile water in aseptic conditions. Immature embryos were isolated and incubated with *Agrobacterium* strain EHA 105 for 5 min. After co-cultivation at 25 °C for 2 d in darkness, the embryonic axes were removed with a scalpel, and the scutella were transferred onto plates. After 5 days, the tissues were then transferred to callus selection medium for 2 weeks. The immature embryos were then placed on induction medium for 3 weeks. The calli were then differentiated under continuous illumination (5000 lx) with fluorescent lights at 25 °C for 14 days. The regenerated shoots were transferred to root elongation medium. The rooted plantlets were then transferred into pots and grown in growth chambers, where they were cultivated at a temperature of 20 °C with light intensity stronger than 60,000 lx, and a night temperature of 16 °C.

### DNA extraction and PCR conditions for Illumina amplicon sequencing

To detect mutations in common wheat protoplasts, wheat protoplasts were collected by centrifugation, and genomic DNA was extracted using the Tiangen DNAquick Plant System (Tiangen, Beijing, China) for deep sequencing. The amplifications of different target genes were carried out using specific primers. Forward and reverse barcodes (Additional file 1: Table S3) were added to the end of the PCR products for library construction. The target regions of three genes corresponding to the *Pinb, waxy* and *DA1* genes were amplified by five pairs of primers with corresponding barcodes. Equal amounts of PCR products were mixed as a pool, and one 2 × 150 bp paired-end library was constructed following the method by German [34]. The samples were used for Illumina sequencing at Novogene company (Beijing, China). Indels, including insertions and deletions, occurring at the targeted sites of the target genes were considered mutations.

To detect mutations in transgenic common wheat seedlings, the target regions of the *DA1* gene were amplified by PCR using A-, B- and D-genome-specific primers (Additional file 1: Table S4). Genomic DNA was isolated from the leaves of the T0 transgenic wheat seedlings. The fragments containing the target site were amplified from individual samples by site-specific primers with the FastPfu polymerase (Transgene, Beijing, China). A PCR-RE assay was then performed with the corresponding restriction enzyme, *Sph*I. The digested and undigested products were separated by electrophoresis on a 2.0% agarose gel. The undigested bands of the target genes were cut and purified, and then subcloned into the pEASY-Blunt vector (Transgene, Beijing, China) and directly Sanger-sequenced. For each sample, at least 6 PCR-positive colonies were randomly selected and sequenced. The mutations were identified by alignment to the reference sequences.

### Bioinformatic analysis

The sequencing data were generated from Illumina HiSeqTM analysis. According to the barcodes and primers, we divided the mixed sequencing data into a raw dataset for each given amplicon. Then, we cleaned the raw data using the FASTX-toolkit (http://hannonlab.cshl.edu/fastx_toolkit/) pipeline to remove low-quality reads, contamination reads, and the adaptor sequences. The reference genes were downloaded from the IWGSC website (http://www.wheatgenome.org/). A reference gene library was constructed and then used for the alignment and mapping of the amplicon gene regions by BWA software [35]. The SAMtools software was used to assess the statistical variation in the results [36]. To show the mutant regions, we assembled the paired-end reads into one sequence with the FLASH package [37], screened the sequences of the target site regions, and calculated the mutant probability using a Perl script.

### Off-target analysis

The potential off-target effects of CRISPR/Cas9 in common wheat were identified using the BLASTN tool against the wheat genome sequence (URGI: https://urgi.versailles.inra.fr/blast/?dbgroup=wheat_all&program=blastn) by searching for the seed sequence plus PAM. Hits with fewer than three mismatches were chosen for amplification and analysis by using specific primers (listed in Additional file 1: Table S5). Specific primers were designed to amplify the potential off-target sites by PCR. The amplicons were subcloned, and 6–8 PCR positive clones were selected for sequencing for each of the genes.

## Results

### Target selection and plasmid construction of the CRISPR/Cas9 system

**Fig. 1:**
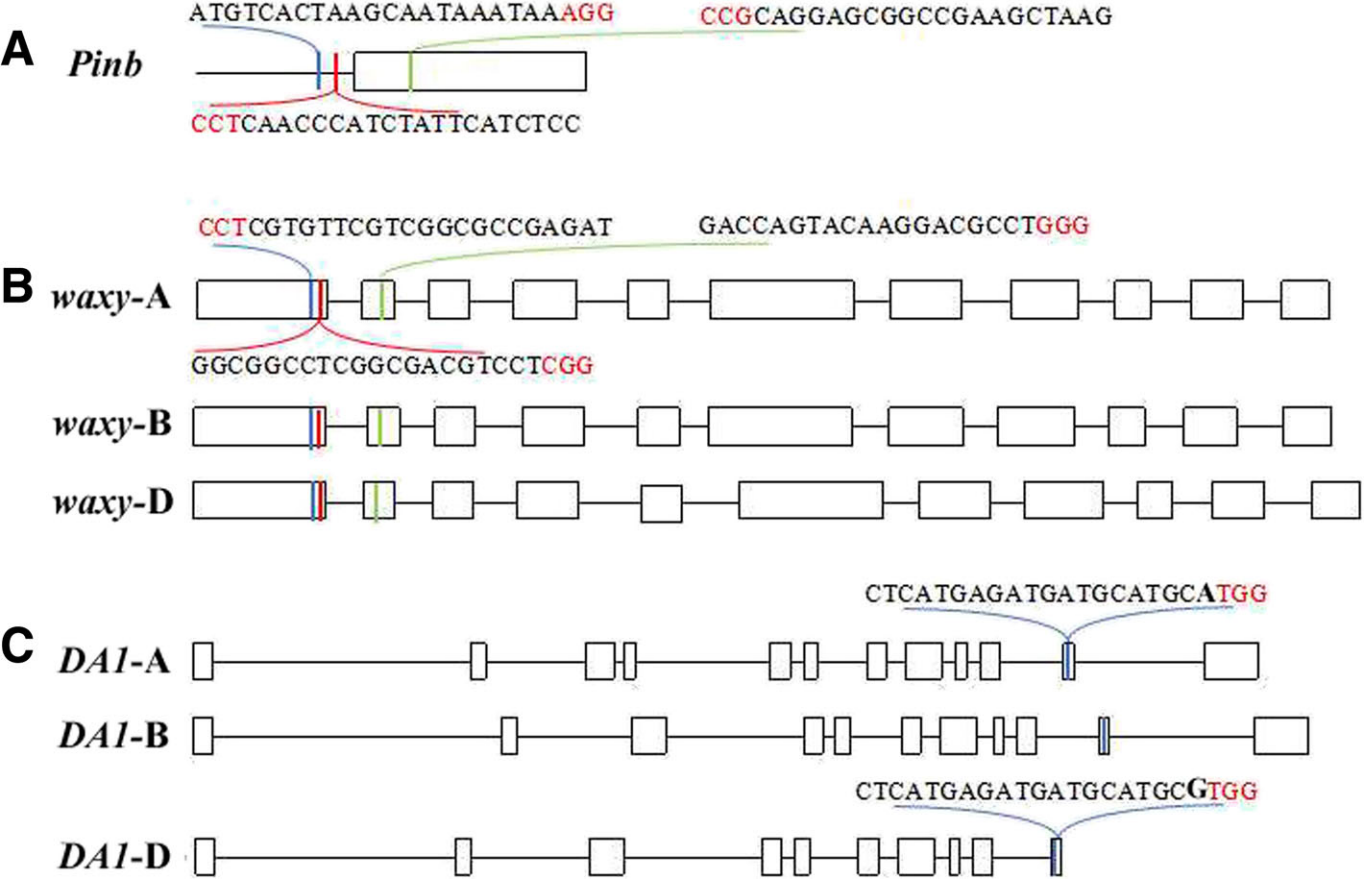
Schematic map of the gRNA target site selection in the target genes. **a** Two sgRNAs (gR1, gR2) and one sgRNA (gR3) of the *Pinb* gene were selected, corresponding to sites in the promoter region and the coding region, respectively. **b** Two sgRNAs (gR1, gR2) and one sgRNA (gR3) of the *waxy* gene were selected, corresponding to sites in the first exon and the second exon, respectively. **c** One sgRNA of the *DA1* gene was selected in the eleventh exon. Introns are shown as lines; exons are shown as boxes

In common wheat (*Triticum aestivum* L.), the *Pinb* gene is located on chromosome 5DS, and no other copy was found in the wheat genome database. Two sgRNAs (gR1 and gR2) were selected in the promoter region up-stream of the ATG start site. One sgRNA (gR3) was designed to recognize the conserved regions in the coding sequence of the *Pinb* gene in hexaploid wheat. Waxy in each genome is encoded by *Wx-A1, Wx-B1* and *Wx-D1*, which are located on chromosomes 7AS, 4AL (translocated from the original locus on 7BS) and 7DS, respectively [29]. The sgRNA targets for *waxy* were designed based on the conserved domains in all three genomes. Two sgRNAs (gR1 and gR2) were designed to target the first exon and one sgRNA (gR3) to target the second exon (Fig. 1b). In addition, the target sites were the common conserved domain of the *Wx-A1, Wx-B1* and *Wx-D1* genes. *DA1* was encoded by three copies located on the 2A, 2B, and 2D chromosomes. The sgRNA of the *DA1* gene (gene accession number: KM005099) was designed on the conserved domains of all three genomes at the eleventh exon, and there was a SNP (A/G) at the sgRNA between the A, B genome and the D genome (Fig. 1c).

These sgRNAs of the target genes were designed using the online tool CRISPRdirect (http://crispr.dbcls.jp/) (Additional file 1: Table S1). In addition, no SNPs were detected in these target sequences compared with the Fielder genome sequence. Two common wheat promoter sequences, TaU6 and TaU3, were used to drive the individual expression of these targets. The constructed expression cassettes were then inserted into the binary vector pYLCRISPR/Cas9Pubi-B using *Bsa*I.

### Targeted mutagenesis of the *Pinb*, *waxy* and *DA1* genes in wheat protoplasts

The protoplast transient expression system is an effective and simple method to evaluate the editing capacity of the CRISPR/Cas9 system for gene editing in wheat. We first confirmed the activity of the CRISPR/Cas9 system in wheat protoplasts by the PEG-mediated transformation method [32]. After 72 h of incubation in darkness at 25 °C, the transformed protoplasts were used for genomic DNA extraction. PCR amplification was performed with barcoded primers. Illumina amplicon sequencing was then performed.

**Fig. 2:**
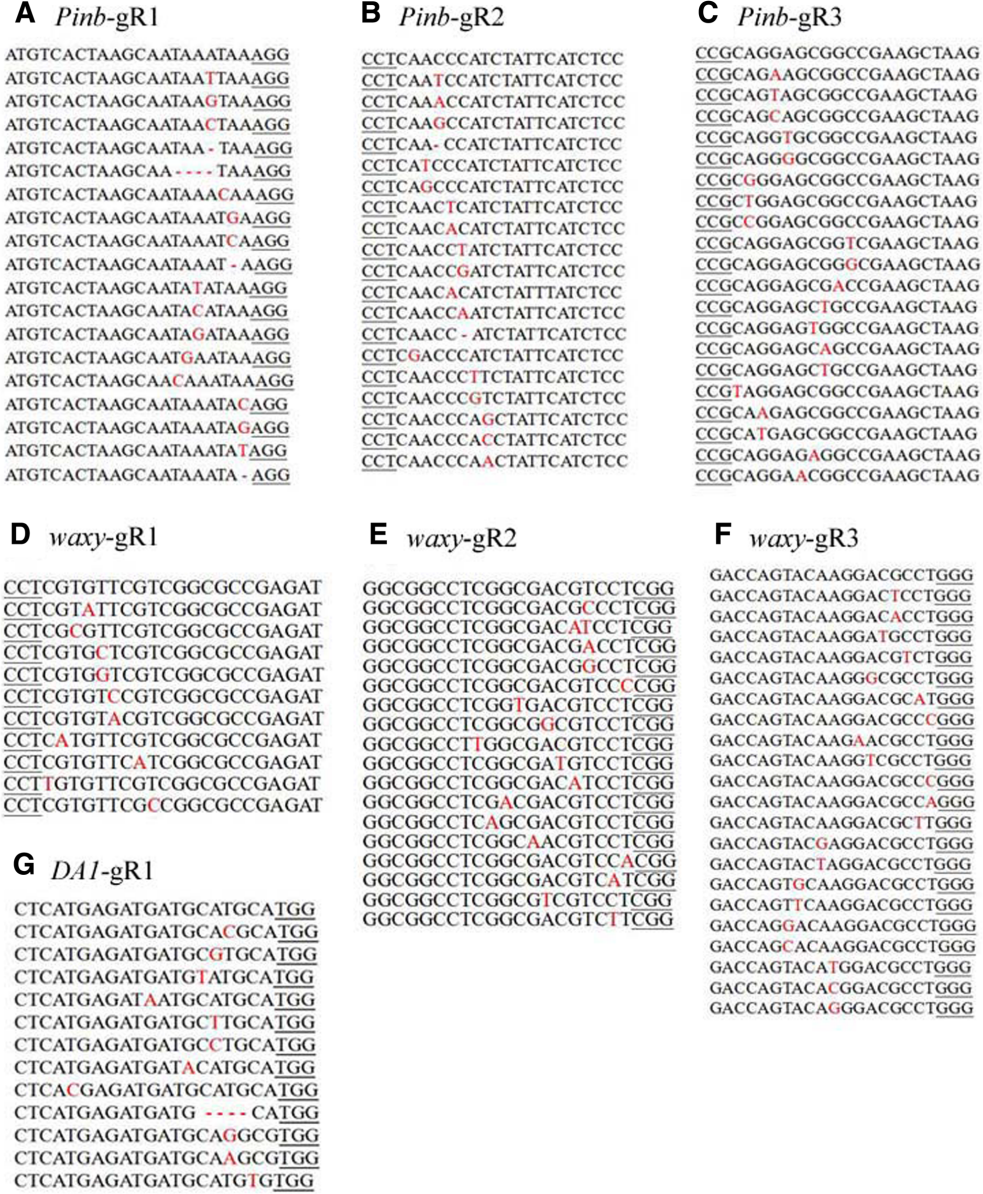
Gene sequences of targeted mutagenesis in wheat protoplasts. Detection of mutations in (**a**) *Pinb*-gR1, (**b**) *Pinb*-gR2, (**c**) *Pinb*-gR3, (**d**) *waxy*-gR1, (**e**) *waxy*-gR2, (**f**) *waxy*-gR3, and (**g**) *DA1*-gR1. The wild-type sequences of the target genes are shown with the PAM sequences underlined in black. Inserted and substituted nucleotides are shown in red. Deletions of nucleotides are shown as dots

The mutant analysis based on the sequencing results is presented in Table 1 and Fig. 2. Mutation frequency ranged from 1.74–6.81%. The targeted edit types were mostly SNPs and indels. SNP frequency was 1.74–6.75%, while lower indel frequencies ranging from 0 to 0.33% were found. Of these, the *Pinb*-gR1 site had a 4.57% mutation efficiency, including 4.49% SNPs and 0.08% indels. At the *Pinb*-gR2 site, the mutation efficiency was 4.37%, including 4.05% SNPs and 0.33% indels. The mutation efficiency at the *Pinb*-gR3 site was 3.86%, including 3.83% SNPs and 0.03% indels. Mutations in the *waxy* gene were less frequent (Table 1), including 1.74% SNPs at gR1, 2.56% SNPs at gR2, and 2.34% SNPs and 0.07% indels at gR3. The *DA1* gene had the highest mutation efficiency at 6.81%, including 6.75% SNPs and 0.05% indels. Of the mutations in the wheat protoplasts, nucleotide substitutions were the major type induced using the CRISPR/Cas9 system. Mutation efficiencies differed between the U6 and U3 promoters and different gene sgRNAs. These results suggested that all the targets could be targeted by the CRISPR/Cas9 system in wheat protoplasts. To ensure higher efficiency of the targeting of Cas9, we chose the sgRNA of the *DA1* gene for further *Agrobacterium*-mediated wheat transformation.

**Table 1:** Illumina sequencing results of targeted mutations in wheat protoplast

Target Name	Clean reads	Mapped information		Target editing information					
		Mapped	%	SNP	%	indel	%	Total	%
		reads							
DA1gR1	462,296	456,040	99.91	30,793	6.75	242	0.05	31,035	6.81
PinbgR12-g1	1,361,836	1,357,466	99.68	60,923	4.49	1094	0.08	62,017	4.57
PinbgR12-g2	1,361,836	1,357,466	99.68	54,913	4.05	4455	0.33	59,368	4.37
PinbgR3	596,786	573,600	96.11	21,982	3.83	178	0.03	22,160	3.86
waxygR12-g1	1,235,538	1,151,644	93.21	20,080	1.74	14	0	20,094	1.74
waxygR12-g2	250,826	164,766	65.69	4208	2.55	6	0	4214	2.56
waxygR3	121,510	120,336	99.03	2819	2.34	79	0.07	2898	2.41

### CRISPR/Cas9-mediated genome editing of the *DA1* gene in T0 transgenic wheat

**Fig. 3:**
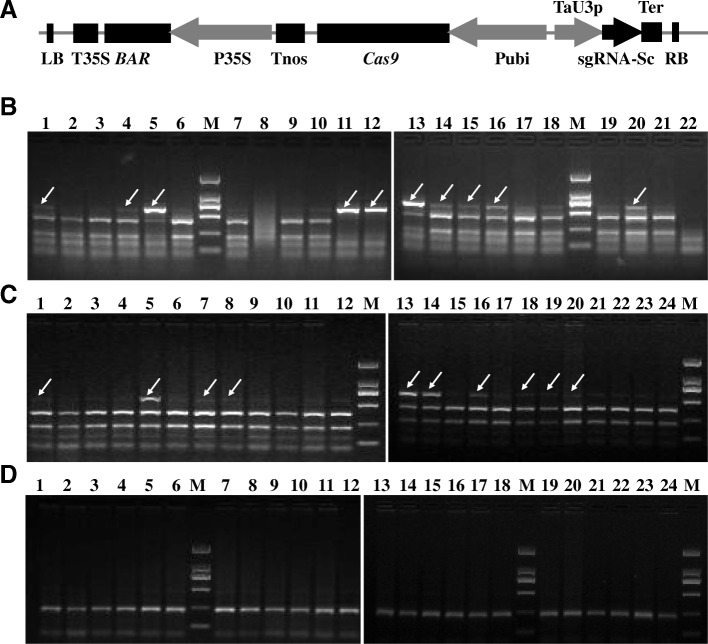
Detection of mutations in T0 *DA1*-editing plants using a PCR-restriction enzyme (PCR-RE) assay. **a** Schematic map of the binary vector of the *DA1* gene for genome editing in wheat. Cas9 is expressed with a ubiquitin promoter. The synthetic guide RNA (sgRNA) is derived using U3 promoters. **b** Gel electrophoresis of PCR products amplified from the mutated region of the A genome with specific primers and digested with *Sph*I. Lanes 1–21 are the digested DNA of the PCR products amplified from different transgenic plants; lane 22 is a wild-type sample. **c** Gel electrophoresis of PCR products amplified from the mutated region of the B genome with specific primers and digested with *Sph*I. Lanes 1–23 are the digested DNA of the PCR products amplified from different transgenic plants; lane 24 is a wild-type sample. **d** Gel electrophoresis of PCR products amplified from the mutated region of the D genome with specific primers and digested with *Sph*I. Lanes 1–23 are the digested DNA of the PCR products amplified from different transgenic plants; lane 24 is a wild-type sample

*Agrobacterium*-mediated transformation was applied in wheat to test whether the CRISPR/Cas9 system could perform gene editing in stable transgenic plants. After transformation of wheat embryos and regeneration of transgenic lines, detection of mutations in the targeted sequence regions was performed in the T0 plants. Twenty-four bialaphos-resistant lines (T0–1 to T0–24) were identified via a PCR-RE assay. Genomic DNA was extracted from the leaves of the T0 plants. In the PCR-RE analysis, mutation detection was performed with primers flanking the designated target sites. To identify mutation types, specific primers were designed to sequence the target regions of the A, B and D genomes. Then, the PCR products were digested by the restriction enzyme *Sph*I for mutation detection. The results representing non-mutation show two completely digested bands. As shown in Fig. 3, undigested bands with putative mutations were found in only the A and B genomes of the transgenic lines, but not in the D genome. Of the 24 bialaphos-resistant plants, a total of 13 mutated lines were identified, a 54.17% frequency of mutagenesis. For A and B genome analysis, there were two different band types on the agarose gel compared with the WT results. Compared with the WT plants, which had two completely digested bands, four lines (T0–6, T0–14, T0–15 and T0–17) showed one larger undigested band, and the other transgenic lines showed one undigested band and two digested bands.

**Fig. 4:**
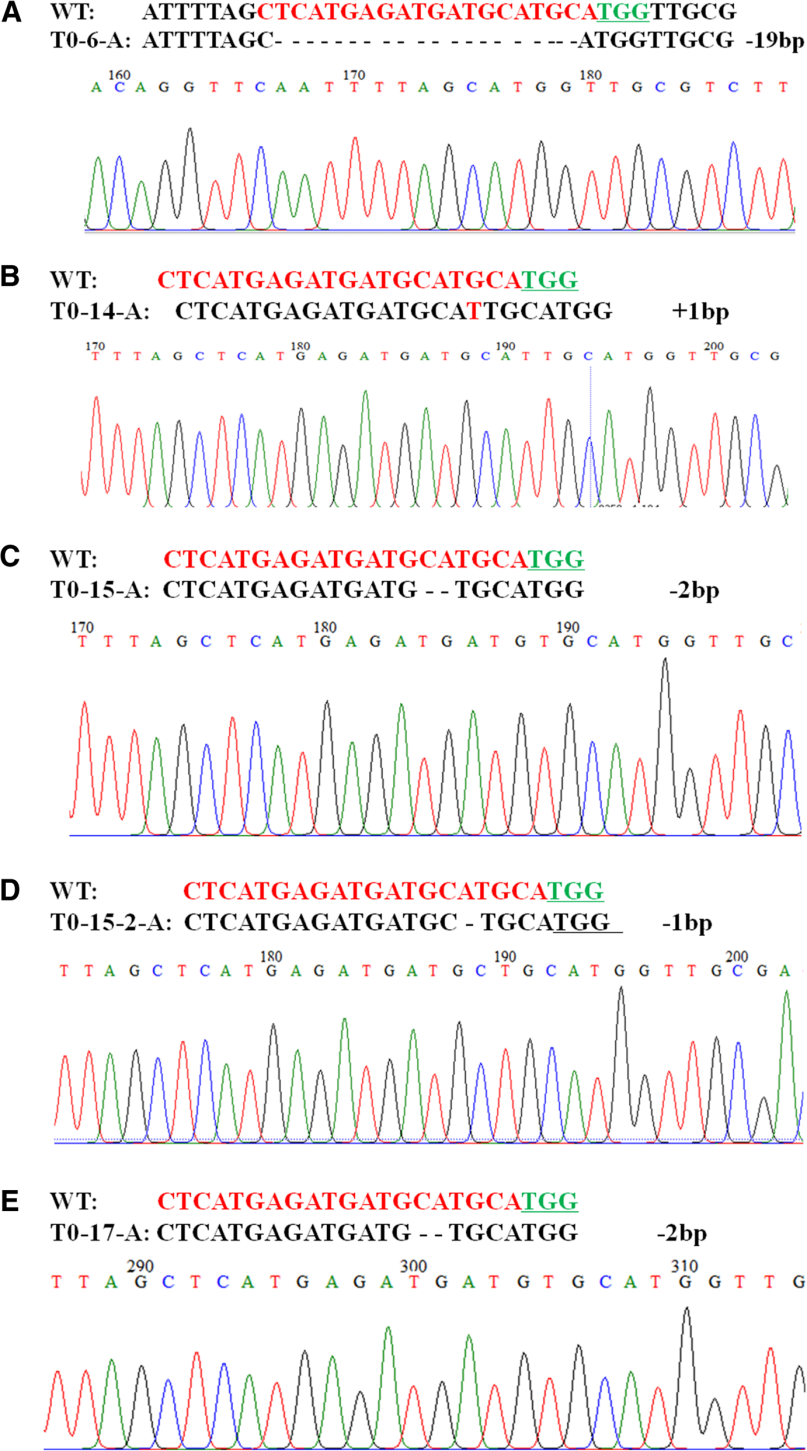
Detailed sequence analysis of CRISPR/Cas9-induced *DA1* gene mutations in the T0 generation. **a-e** Targeted mutagenesis in the A genomes of selected T0 plants with site-specific mutations accompanied by the corresponding regions of the sequencing chromatograms. The nucleotides of the target site are in red. Green underlined nucleotides indicate the PAM sequences of the sgRNA. “-” and “+” indicate the deletion and insertion of the indicated numbers of nucleotides, respectively

**Fig. 5:**
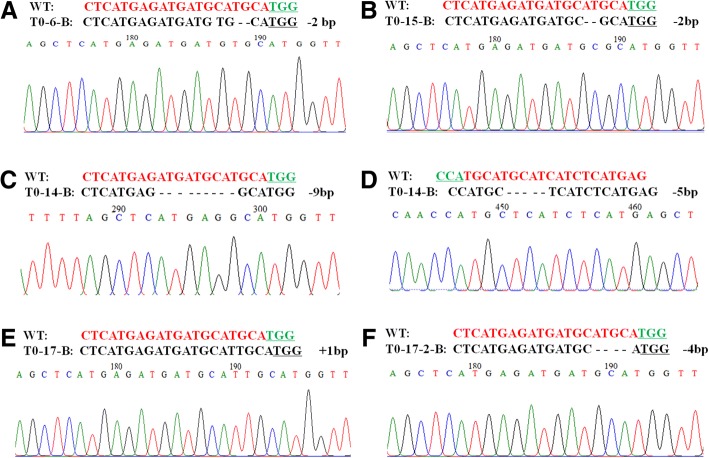
Detailed sequence analysis of CRISPR/Cas9-induced *DA1* gene mutations in the T0 generation. **a-f** Targeted mutagenesis in the B genomes of selected T0 plants with site-specific mutations accompanied by the corresponding regions of the sequencing chromatograms. The nucleotides of the target site are in red. Green underlined nucleotides indicate the PAM sequences of the sgRNA. “-” and “+” indicate the deletion and insertion of the indicated numbers of nucleotides, respectively

The four transgenic lines (T0–6, T0–14, T0–15 and T0–17) with higher mutation efficiency were selected for further Sanger sequencing. The undigested bands were subcloned and Sanger-sequenced to detect the sequence alterations. Six to ten clones for each sample were sequenced. Sequence analysis indicated that the above four lines all had mutations in the A and B genomes. Different bases, insertions and deletions were found in the target region. In the A genome (Fig. 4), line T0–6 had a 19 bp deletion, line T0–14 had a 1 bp insertion, and line T0–15 had two edit types, including a 1 bp deletion and a 2 bp deletion. In the B genome (Fig. 5), line T0–6-B had a 2 bp deletion; line T0–15B also had a 2 bp deletion; T0–14 had two types of mutation, including a 1 bp insertion and 4 bp deletion; and T0–17 also had two kinds of mutation, a 1 bp insertion and a 4 bp deletion. Therefore, our results demonstrate that the constructed CRISPR/Cas9 vector can efficiently achieve targeted mutagenesis in the wheat genome.

### Off-target potential was not detected

We further assessed off-target effects using the *DA1* gene of the CRISPR/Cas9 construct. The off-target potential analysis was performed by analysing whether mutations occurred at other sgRNA homologous positions in the common wheat genome. To detect off-target events, four putative off-target sites that could produce mutations were selected for further study (Additional file 1: Table S5). The primers used for amplifying the potential off-target areas are listed in Additional file 1: Table S5. The sequencing results verified that no mutations were found in the PCR products from these potential sites. These results suggested that the off-target effect can be neglected. Therefore, the CRISPR/Cas9 system was highly specific for targeted mutation in the *DA1* gene.

## Discussion

In recent years, the CRISPR/Cas9 system has developed rapidly [38]. Common wheat (*Triticum aestivum* L.) is one of the most important crops in the world and has a complex genome consisting of three different genomes: A, B, and D [39, 40]. The hexaploid structure of the wheat genome means that the investigation and optimization of the genome editing system is a challenging research objective. In common wheat, this technique has been shown to be effective in both protoplasts and transgenic plants [22, 24, 32]. However, the reported transformations of transgenic wheat plants with the CRISPR/Cas9 system have used biolistic-mediated methods. *Agrobacterium tumefaciens*-mediated and biolistic (gene gun)-mediated methods are two major and effective transformation methods. Compared to the biolistic method, the *Agrobacterium tumefaciens*-mediated transformation is more popular, because it not only usually inserts single or few copies of transgenes in plants but also does not require an expensive particle gun apparatus and supplies. In the present study, we first verified the targeting efficiency of CRISPR/Cas9 via protoplast transfection in vivo and then proceeded to generate transgenic wheat plants by *Agrobacterium*-mediated transformation. This study is an application of targeted mutagenesis with CRISPR/Cas9 in wheat by the *Agrobacterium tumefaciens*-mediated method.

The wheat protoplast transient expression system is an effective and simple method to evaluate the gene editing capacity of the CRISPR/Cas9 system. In this study, we applied the CRISPR/Cas9 system to three genes: *Pinb, waxy* and *DA1*. Seven target sites of these three genes were chosen to construct expression vectors. The targeted mutagenesis caused by the CRISPR/Cas9 constructs was confirmed by Illumina sequencing. The transient expression of CRISPR/Cas9 was detected in wheat protoplasts successfully. Combination of these results showed that the mutation efficiency of *DA1*-gR1 was the highest (6.8%) compared to the other targets (1.27–4.57%). The difference may be due to the location sites and GC contents of the chosen sgRNAs. The mutation efficiency in our study was lower than those in other reports. In the report of wheat mutagenesis via CRISPR/Cas9 by Kim et al. [24] in wheat protoplasts, the mutation efficiency for *TaDREB2* was 6.7%, while that for *TaERF3* was 10.2%. In addition, in maize mutagenesis via CRISPR/Cas9 targeting the *ZmIPK* gene, one gRNA achieved 16.4% mutation frequency, and that of the other gRNA was 19.1% in mesophyll protoplasts [12]. A higher efficiency of 40.7–52.0% was achieved in watermelon protoplast cells, while 38.5% was found in *Nicotiana benthamiana* protoplasts [2]. The low editing efficiency in this study might be due to the large and complex genome of wheat and the fragile nature of wheat protoplasts [41]. The choice of targeting sites is the critical step in using the CRISPR/Cas9 technology. It has been reported that the GC content of the sgRNA targeting sequence is important for the efficiency of CRISPR/Cas9 [22]. In our study, the sgRNAs had a range of GC% content of 27–76%; those having a higher GC% content, such as *waxy*-gR2 (76%), did not show a higher editing efficiency (2.56%), and those having a lower GC% content (*Pinb*-gR1 with 27%) had an editing efficiency of 4.57% (Table 1).

The mutation types and frequencies may have relationships with a variety of factors. The main factors may be the CRISPR/Cas9 construct transformation method, which could cause different transgene copy numbers. The transformation efficiency in most wheat cultivars was lower than that in other crops. Successful and efficient T-DNA insertion by *Agrobacterium*-mediated transformation has been achieved in wheat [42]. In this study, transgenic plants could be obtained in three months with transformation efficiencies up to 80%. Mutations produced by the CRISPR/Cas9 system in T0 transgenic wheat plants could be detected. In addition, the mutation efficiency reached 54.17%, much higher than the 5.6% previously reported in wheat [22]. The reported mutagenesis efficiencies differ significantly among different species and different CRISPR/Cas9 platforms. In rice, mutagenesis rates could reach 100% in many labs [5, 6, 43]. CRISPR/Cas9 systems could produce more than 50% mutated T0 plants in tomato and soybean [16, 44]. In maize, 2–100% of T0 plants were reported [14, 15, 45, 46]. Furthermore, the sequences and chromosomal locations of the target sites may affect the Cas9/gRNA ribonucleoprotein’s access to the target sites [47, 48].

Of course, the specificity of CRISPR/Cas9 genome editing also requires consideration during genome editing because of the occurrence of random mutations [49, 50] . There is still a high possibility of off-target mutations in wheat because of its complex polyploid genome structure [51]. The genome editing specificity may decrease particularly for genes that possess high copy numbers in several genomic locations. In this study, transgenic T0 plants of the *DA1* gene showed successful editing in the A and B genomes, but not in the D genome. There is a mismatch between the A, B genome and the D genome. The one mismatch of the D genome maybe the main result of not editing the D allele. This just illustrates the accuracy of the method proposed in this paper from another aspect. Furthermore, there were no off-target mutations found in the highly homologous sequences.

Because of the large and complex genome of wheat, the detection method of transgenic mutagenesis was relatively labour-intensive. Wheat has three different genomes: A, B, and D. Therefore, the detection method becomes an important issue. For the PCR-RE assay, it was necessary to design specific primers to distinguish the A, B and D genomes, and an appropriate restriction enzyme was also needed. However, it was too difficult and time-consuming to design and verify specific primers to distinguish the A, B and D genomes. In addition, the target sequence was not necessarily suitable for the selection of a restriction enzyme. In our study, one of the most important reasons to choose the *DA1* gene for transgenic analysis was that specific primers for the A, B and D genomes had been designed and found to work well in a previous gene function analysis. Therefore, Illumina sequencing was certainly considered in the detection of transgenic wheat mutations.

The application of the CRISPR/Cas9 system would be an important part of crop improvement. For wheat breeding, in which transgenic technologies are available, gene targeting of key functional genes would become a promising biotechnological strategy. In this study, the CRISPR/Cas9 system could edit target genes efficiently in wheat by *Agrobacterium*-mediated stable transformation. Our results confirmed that the CRISPR/Cas9 system can be used as a promising tool for facilitating wheat breeding. In addition, it still requires further investigation of inheritance in the mutated transgenic lines and the relationship between the mutation types and the target genes. The selected mutant T0 lines were self-pollinated, and a transmission analysis of the mutations must also be performed in the T1 and T2 progeny.

It had reported an efficient DNA-free genome editing method of bread wheat using CRISPR/Cas9 ribonucleoprotein complexes [52]. Our method could obtain DNA-editing non-transgenic material and used for conventional breeding. The continuous expressing Cas9 gene could produce more abundant mutation types in the transgenic plants and their progeny. Transgenes (Cas9, sgRNA, and so on) can be eliminated from wheat genomes following screening of segregating populations. This segregation of CRISPR/Cas9 transgenes from mutations of interest can result in non-transgenic mutant plant progeny. The selected material could be backcrossed to the adapted and high yielding wheat varieties for conventional wheat breeding.

## Conclusions

The CRISPR/Cas9 system has been widely used in editing plant genomes. Because the genetic transformation of wheat is difficult, the CRISPR/Cas9 system in wheat is relatively less studied and were all used the biolistic transformation method. In our paper, we efficiently applied targeted mutagenesis in common wheat protoplasts and transgenic T0 plants using the CRISPR/Cas9 system. And transgenic wheat plants obtained by *Agrobacterium*-mediated transformation had higher mutagenesis efficiency of 54.17%. Our results showed that the CRISPR/Cas9 system could edit target genes efficiently in wheat by *Agrobacterium*-mediated stable transformation. The CRISPR/Cas9 system can be used for targeted mutations and facilitating wheat genetic improvement.

## Additional file

Additional file 1: **Table S1.** sgRNA selection. **Table S2.** Primers for vector construction. **Table S3.** Primers and barcodes for Illumina sequencing. **Table S4.** Specific A, B and D genome primers of the *DA1* gene for target sites amplification. **Table S5.** Off-targeting of the designed sgRNA for the *DA1* gene. (ZIP 16 kb)
