# Supplementary material for: Targeted mutagenesis using the Agrobacterium tumefaciens-mediated CRISPR-Cas9 system in common wheat
Source: BMC Plant Biol. 2018 Nov 26;18:302. doi: 10.1186/s12870-018-1496-x (PMC6258442; doi:10.1186/s12870-018-1496-x)
Supplement: Supplementary file 1 — Table S1. sgRNA selection. Table S2. Primers for vector construction. Table S3. Primers and barcodes for Illumina sequencing. Table S4. Specific A, B and D genome primers of the DA1 gene for target sites amplification. Table S5. Off-targeting of the designed sgRNA for the DA1 gene. (ZIP 16 kb) [file 12870_2018_1496_MOESM1_ESM.zip › Additional file1 Table S1.doc]

Additional file1: Table S1 sgRNA selection

|  | sgRNA sequence(5’-3’) | GC content (%) |
| --- | --- | --- |
| Pinb-TaU6-gR1 | GATGTCACTAAGCAATAAATAA | 27% |
| Pinb-TaU6-gR2 | GGAGATGAATAGATGGGTTG | 45% |
| Pinb-TaU6-gR3 | GCTTAGCTTCGGCCGCTCCTG | 67% |
| DA1-TaU3-gR1 | ACTCATGAGATGATGCATGCA | 43% |
| waxy-TaU3-gR1 | ATCTCGGCGCCGACGAACACG | 67% |
| waxy-TaU3-gR2 | AGGCGGCCTCGGCGACGTCCT | 76% |
| waxy-TaU3-gR3 | AGACCAGTACAAGGACGCCT | 55% |
