# Supplementary material for: Targeted mutagenesis using the Agrobacterium tumefaciens-mediated CRISPR-Cas9 system in common wheat
Source: BMC Plant Biol. 2018 Nov 26;18:302. doi: 10.1186/s12870-018-1496-x (PMC6258442; doi:10.1186/s12870-018-1496-x)
Supplement: Supplementary file 1 — Table S1. sgRNA selection. Table S2. Primers for vector construction. Table S3. Primers and barcodes for Illumina sequencing. Table S4. Specific A, B and D genome primers of the DA1 gene for target sites amplification. Table S5. Off-targeting of the designed sgRNA for the DA1 gene. (ZIP 16 kb) [file 12870_2018_1496_MOESM1_ESM.zip › Additional file1 Table S2.doc]

Additional file1: Table S2 Primers for vector construction

|  | Oligo sequence (5’-3’) |
| --- | --- |
| Pinb-TaU6-gR1F | CTTGGATGTCACTAAGCAATAAATAA |
| Pinb-TaU6-gR1R | AAACTTATTTATTGCTTAGTGACATC |
| Pinb-TaU6-gR2F | CTTGGGAGATGAATAGATGGGTTG |
| Pinb-TaU6-gR2R | AAACCAACCCATCTATTCATCTCC |
| Pinb-TaU6-gR3-F | CTTGGCTTAGCTTCGGCCGCTCCTG |
| Pinb-TaU6-gR3-R | AAACCAGGAGCGGCCGAAGCTAAGC |
| DA1-TaU3-gR1-F | AAGCACTCATGAGATGATGCATGCA |
| DA1-TaU3-gR1-R | AAACTGCATGCATCATCTCATGAGT |
| waxy-TaU3-gR1-F | AAGCATCTCGGCGCCGACGAACACG |
| waxy-TaU3-gR1-R | AAACCGTGTTCGTCGGCGCCGAGAT |
| waxy-TaU3-gR2-F | AAGCAGGCGGCCTCGGCGACGTCCT |
| waxy-TaU3-gR2-R | AAACAGGACGTCGCCGAGGCCGCCT |
| waxy-TaU3-gR3-F | AAGCAGACCAGTACAAGGACGCCT |
| waxy-TaU3-gR3-R | AAACAGGCGTCCTTGTACTGGTCT |
| 131-F | TCTAGAGGTCTCGCTCGTGACCAAGCCCGTTATTC |
| 131-R | CTCGAGGGTCTCCACCGAAGCTTATCGATACCCTC |
| 132-F | TCTAGAGGTCTCCAAGCCATGAATCCAAACCACACG |
| 132-R | CTCGAGGGTCTCGACCGGCTTCTTGGTGCCGCGCCT |
