# Supplementary material for: Targeted mutagenesis using the Agrobacterium tumefaciens-mediated CRISPR-Cas9 system in common wheat
Source: BMC Plant Biol. 2018 Nov 26;18:302. doi: 10.1186/s12870-018-1496-x (PMC6258442; doi:10.1186/s12870-018-1496-x)
Supplement: Supplementary file 1 — Table S1. sgRNA selection. Table S2. Primers for vector construction. Table S3. Primers and barcodes for Illumina sequencing. Table S4. Specific A, B and D genome primers of the DA1 gene for target sites amplification. Table S5. Off-targeting of the designed sgRNA for the DA1 gene. (ZIP 16 kb) [file 12870_2018_1496_MOESM1_ESM.zip › Additional file1 Table S3.doc]

Additional file1: Table S3 Primers and barcode for Illumina sequencing

|  | Primers (5’-3’) | Barcode |
| --- | --- | --- |
| DA1-1-5F | **ATCACG**AACCTGATGAACTCTGAATGTG | ATCACG |
| DA1-1-5R | **TTAGGC**GGTTGTCTTTCATTCTTGTGG | TTAGGC |
| Pinb-gR12-F1 | **CAGATC**AGCATTAGCCAAAGCAAC | CAGATC |
| Pinb-gR12-R1 | **ACTTGA**CGCTACAAGAGCAAGGAGA | ACTTGA |
| Pinb-gR3-F1 | **TAGCTT**TGTAGCGAGCACAACCTT | TAGCTT |
| Pinb-gR3-R1 | **GGCTAC**GTGAAACATCGCTCCATC | GGCTAC |
| waxy-gR12-F1 | **AGTTCC**TGCCTCTCCATGGTGGTGCG | AGTTCC |
| waxy-gR12-R1 | **ATGTCA**CAAGCTTACGGCCATGGC | ATGTCA |
| waxy-gR3-F1 | **CCGTCC**CACTGCCTTCTTATAAATGTTTC | CCGTCC |
| waxy-gR3-R1 | **GTAGAG**TGCAGAAATGTGCAGGAGCATG | GTAGAG |
