# Supplementary material for: Targeted mutagenesis using the Agrobacterium tumefaciens-mediated CRISPR-Cas9 system in common wheat
Source: BMC Plant Biol. 2018 Nov 26;18:302. doi: 10.1186/s12870-018-1496-x (PMC6258442; doi:10.1186/s12870-018-1496-x)
Supplement: Supplementary file 1 — Table S1. sgRNA selection. Table S2. Primers for vector construction. Table S3. Primers and barcodes for Illumina sequencing. Table S4. Specific A, B and D genome primers of the DA1 gene for target sites amplification. Table S5. Off-targeting of the designed sgRNA for the DA1 gene. (ZIP 16 kb) [file 12870_2018_1496_MOESM1_ESM.zip › Additional file1 Table S4.doc]

Additional file1: Table S4 Specific A, B and D genome primers of *TaDA1* gene for target sites amplification

|  | Primers(5’-3’) |
| --- | --- |
| CPF-A-F | CTACGGATTTACTCAAGGGAGCTAATGAGCG |
| CPF-A-R | CGCAACTTCTGGTAAAAATCTTGAAACATGAC |
| CPF-B-F | CTACGAGTTTAATCAAGGGAACTAATGTGCA |
| CPF-B-R | GCACTAGCAGATTCATTACAAAATCTAATAG |
| CPF-D-F | GAAGATATTCTGGGTTACAGAAC |
| CPF-D-R | ATAATTCCACTGATGTAAGACCG |
