# Supplementary material for: Targeted mutagenesis using the Agrobacterium tumefaciens-mediated CRISPR-Cas9 system in common wheat
Source: BMC Plant Biol. 2018 Nov 26;18:302. doi: 10.1186/s12870-018-1496-x (PMC6258442; doi:10.1186/s12870-018-1496-x)
Supplement: Supplementary file 1 — Table S1. sgRNA selection. Table S2. Primers for vector construction. Table S3. Primers and barcodes for Illumina sequencing. Table S4. Specific A, B and D genome primers of the DA1 gene for target sites amplification. Table S5. Off-targeting of the designed sgRNA for the DA1 gene. (ZIP 16 kb) [file 12870_2018_1496_MOESM1_ESM.zip › Additional file1 Table S5.doc]

Additional file1: Table S5 Off-targeting of designed sgRNA for *TaDA1* gene

|  | Genomic location | Off-target region | Primers | Mutation  Occurence |
| --- | --- | --- | --- | --- |
| DA1-Offtarget1 | ta_iwgsc_5as_v1_1510927 | CCACGCACGCATCATCTCATGAG | F:5’-TACAGAAACGCACCCGTGAT-3’  R:5’-GCCCTGTTTCAACTGAATTGG-3’ | No |
| DA1-Offtarget2 | ta_iwgsc_2as_v1_5164109 | CCAGGCTTGCATCATCTCATGAG | F:5’-AGGAATTCCAAAGCCTGACC-3’  R:5’-TAACAATGCCAGAATCTCCAGC-3’ | No |
| DA1-Offtarget3 | ta_iwgsc_5bl_v1_5393123 | CCATGGCATGCACATTCATGAG | F:5’-TAGCATCGTTAGCGGTAGACAC-3’  R:5’-GCAAGCCGCCACTTCTATTTT-3’ | No |
| DA1-Offtarget4 | ta_iwgsc_2ds_v1_5389115 | CCACGCATGCATCATCTCATGAG | F:5’-TTTGGCCCTGACTTCTAAGC-3’  R:5’-TATGGTCTCCCAAGGTGAAGC-3’ | No |
